# Supplementary material for: Buruli ulcer surveillance in south-eastern Australian possums: Infection status, lesion mapping and internal distribution of Mycobacterium ulcerans
Source: PLoS Negl Trop Dis. 2024 Nov 5;18(11):e0012189. doi: 10.1371/journal.pntd.0012189 (PMC11581399; doi:10.1371/journal.pntd.0012189)
Supplement: S1 Appendix — S1.1 Examination record sheet for adult and sub-adult possums examined during the trapping studies. S1.2 Examination record sheet for back young examined during the trapping studies. S1.3 Final examination record sheet for possums necropsied during the study. The study protocol and record sheet were amended to add some tissues (heart, brain and bone marrow/femur) after the study had commenced. (PDF) [file pntd.0012189.s001.pdf]

EXAM RECORD: Possum trapping- *adults*

Beating Buruli in VIC

Date:________________ **Operators:**___________________
Trap #:_______________ **Bag #:**__________________
Address:____
Trap location:____
Status: [ ] 1st exam [ ] Seen previously
Demeanour: [ ] Alert [ ] Depressed [ ] Aggressive
Comments:

Possum ID: T________

Species: [ ] Ringtail [ ] Brushtail **Sex:** [ ] Male [ ] Female

Microchip sticker:

Weight of possum:__________________ Weight (bagged possum):____________________ Weight (bag):______________________

Anaesthesia/sedation & examination record

[ ] Gas anaesthesia (iso in 1-1.5mL/min O_2 via face mask)
Time of induction: _____:_____
Induction: [ ] 5% iso [ ] Other:__________________
Maintenance: [ ] 2% iso [ ] Other:__________________

[ ] IM sedation (Zoletil-100 at 10mg/kg BW; 0.1mL for 1kg possum)
Time administered: ______:______
Dose given:______mL Needle: [ ] 25G [ ] Other:_____G
IM location: [ ] Rump- L / R (circle) [ ] Other:_______________

Time examination started: ______:______ **Warming method:** [ ] Electric heat pad [ ] Hot water bottle

Time	Iso %	O_2 mL/min	HR	RR	Temp °C	Comments
:						
:						
:						
:						
:						
:						

Age class: [ ] Adult [ ] Sub-adult
Health status: [ ] Normal [ ] Abnormal
BCS: ___________ (1 Emaciated <-> 5 Obese)
Ectoparasites present? [ ] No [ ] Yes
Ulcerations present? [ ] No [ ] Yes

Joeys present? [ ] No [ ] Yes- **JV**________________________________ (Joey ID/s) ; [ ] Joey exam sheet/s completed

ID marking performed: [ ] Microchip [ ] Ear tattoos- code:____________ L / R (circle) [ ] Collar- colours:___________________
Notes:____

Samples collected – standard:
[ ] **Faeces** [ ] Ziplock [ ] Cryovials (for Alistair) [ ] **Swabs** [ ] Oral [ ] Cloacal [ ] Pouch [ ] **Photos** [ ] Dorsal [ ] Ventral
[ ] **Blood** [ ] Lith-Hep (green) [ ] EDTA (purple) Vol:_____mL ; _____#vials Vein: [ ] Lateral tail- L / R (circle) [ ] Ventral tail

Additional samples if applicable [ ] Other:____
[ ] **Ectoparasites** (in 70-80% ethanol): [ ] Fleas [ ] Other:_______________________________
[ ] **Ulcerative lesions**: [ ] Swab [ ] Swab cyto prep [ ] Photos [ ] Mapping silhouette* (see next page)

Exam findings:

Tooth wear notes* (see next page):

Repro notes* (F= pouch condition; M= scent gland development, scrotal width):

ABs/fluids given? [ ] No [ ] Yes:_______________________________________(brand name, dose, route)
Time exam finished/iso turned off: ______:______ **Exam duration:**________________________mins
Time O_2 turned off: ______:______ **Time returned to bag:** ______:______ **Time fruit given:** ______:______

Anaesthetic/sedation depth: [ ] Light [ ] Surgical [ ] Deep **Complications:** [ ] None [ ] Minor [ ] Serious [ ] Fatal
Recovery: [ ] Normal [ ] Abnormal [ ] Prolonged [ ] Violent [ ] Died during recovery

Outcome: [ ] Back to wild Time released: ______:______ Comments:_____________________________
[ ] Taken to vet Clinic name:______________________________ Time admitted: ______:______
[ ] Euthanised [ ] **Anaesthetised** (isoflurane) [ ] **Sedated** (IM Zoletil-100) ____________________(dose & location)
Vol Lethabarb given (1mL/2kg BW):______________mL Route & location:_______________________________
Time Lethabarb given: ______:______ Time of death: ______:______ [ ] AWO notified? ________________(date & method)
Additional comments:

Signed: In RedCap? [ ]

EXAM RECORD: Possum trapping- *joeys*

Beating Buruli in VIC

Date:____________________ **Operators:**______________________________
Trap #:_______________ **Bag # (mother):**______________________________
Address:____
Trap location:____

Mother's ID: T_______ **Mother's microchip #:**_____________________________

Mother's status: [ ] 1st exam [ ] Seen previously
Mother's demeanour: [ ] Alert [ ] Depressed [ ] Aggressive
Mother's health status: [ ] Normal [ ] Abnormal
Mother's restraint method: [ ] Anaesthetised (isoflurane) [ ] Sedated (Zoletil-100)
Comments:

Joey ID: JT_____
Species: [ ] Ringtail
[ ] Brushtail
Joey type: [ ] Pouch
[ ] Back
Joey sex: [ ] Male
[ ] Female
[ ] Undetermined

Joey observation/examination record

POUCH JOEY: Observe only; do not handle.

Attached to teat? [ ] Yes [ ] No
Eyes open? [ ] Yes [ ] No
Tips of ears free? [ ] Yes [ ] No
Facial whisker development? [ ] Yes [ ] No
Fur development: [ ] None ('pinky') [ ] Slight fur tinge
[ ] Medium fur [ ] Well-furred

Joey's demeanour: [ ] Alert [ ] Passive [ ] Active
Joey's health status: [ ] Normal [ ] Abnormal

Comments:

Samples collected: [ ] Photos (only)

BACK JOEY: Restrain within separate cotton bag.

Time examination started: ______:______
Bag # (joey):______________________________

Weight of joey:______________________g
Weight (bagged joey):____________________g
Weight (bag):_________________________g

Joey's demeanour: [ ] Alert [ ] Passive [ ] Active
Joey's health status: [ ] Normal [ ] Abnormal

Comments:

Head length (mm): Head width (mm):

Samples collected: [ ] Oral swab [ ] Photos
[ ] Other:______________________________________

Estimated age of joey (Refer joey development tables)**:**____days / months **(circle)**

Time exam finished: ______:______ **Exam duration:**________________mins

Joey's outcome: [ ] Stayed in pouch Comments:____
[ ] Returned to bag with mother Bag #:______________________ Time returned to bag: ______:______
[ ] Euthanised [ ] **Anaesthetised** (isoflurane) [ ] **Sedated** (IM Zoletil-100) ___________________________________(dose & location)
Vol Lethabarb given (1mL/2kg BW):______________mL Route & location:______________________________________
Time Lethabarb given: ______:______ Time of death: ______:______ [ ] AWO notified? _________________ (date & method)

Mother's recovery: [ ] Normal [ ] Abnormal [ ] Prolonged [ ] Violent [ ] Died during recovery
Mother's outcome: [ ] Back to wild [ ] Taken to vet [ ] Euthanised

Mother & joey successfully returned to wild? [ ] Yes [ ] No:_______________________________________

Additional comments:

Signed:

EXAM RECORD: Possum necropsy

Beating Buruli in VIC

Specimen ID: N____/____

Extra ID?______________________________

Path ID:______________________

Date of PM: ____________________
Pathologist: ____________________
Time PM started: ______________

Possum details **Where from?**______________________________________
Date of death:______________________ [ ] Died/Found dead [ ] Euth'd [ ] Unknown
History:

Post mortem examination

Weight of possum: __________________ Weight (bagged possum): _______________ Weight (bag): __________________

Carcase state: [ ] Thawed [ ] Fresh **Carcase condition:** [ ] Excellent [ ] Good but some deterioration [ ] Very deteriorated

Species: [ ] Ringtail [ ] Brushtail
Age class: [ ] Adult [ ] Sub-adult [ ] Juvenile / joey
Sex: [ ] Female [ ] Male
Joeys present? [ ] No [ ] Yes- _____ #Back joeys _____ #Pouch joeys

ID markings present? [ ] No [ ] Yes- [ ] Microchip [ ] Ear tattoos- code:______________ L / R (circle) [ ] Collar- colours: ______________________
Details:____

Health status: [ ] Normal [ ] Abnormal
BCS: _______________ (1 Emaciated <--> 5 Obese)
Ectoparasites present? [ ] No [ ] Yes
Ulcerations present? [ ] No [ ] Yes

Systems examination

1. Skin & sensory [ ] NAD [ ] AD
(incl. eyes, nose, brain)
2. Musculoskeletal [ ] NAD [ ] AD
3. Cardiovascular [ ] NAD [ ] AD
4. Respiratory [ ] NAD [ ] AD
5. Digestive [ ] NAD [ ] AD
6. Urogenital [ ] NAD [ ] AD
(No / Abnormality Detected)

Repro status & notes* (see refs next page):
Males: Scent gland activity (*brushtails only*):
[ ] None/low [ ] Med [ ] High

Females: [ ] Non-parous [ ] Parous [ ] Pregnant
[ ] Pouch young present [ ] Lactating [ ] Post-lactation

Tooth wear notes* (see refs next page):

Findings:

Samples collected- standard

STANDARD PLAIN SWABS (-20°C)	✓
Oral cavity	
Cloaca	
Pouch	

OTHER	✓
Photos	
Pouch joeys	

STANDARD TISSUE SAMPLES	10% buff. Formalin	Cryo vial @ -80°C
Ear	N/A	
Nose/muzzle	N/A	
Front footpad	N/A	
Hind footpad	N/A	
Body cavity fluid	N/A	
Submandib. LN		
Heart		
Lung		
Liver- L & R lobes		
Spleen		

STANDARD TISSUE SAMPLES	10% buff. Formalin	Cryo vial @ -80°C
Kidney		
Adrenal gland		
Stomach		
Small intestine		
Caecum		
Large intestine		
Gut contents	N/A	
Faeces	N/A	
Brain		N/A
Femur- upper 1/3		N/A

Samples collected- if applicable

ULCERATIONS	✓
Plain swab/s @-20°C n=________________	
Excised lesion/s @formalin n=_____________	
Excised lesion/s @-80°C n=_______________	
Photographs- w & w/out ruler	
Mapping silhouette* (next page)	

PARASITES (70% EtOH)	✓
Ecto:_____________________________	
Endo:_____________________________	

Time PM finished: ________________
[ ] In APD [ ] In spreadsheet [ ] Scanned
[ ] Photos downloaded ([ ] In RedCap?)

Mapping silhouette for ulcerative lesions

NB: For multiple lesions, number according to body part, eg RF1, RF2 (right fore); S1 (scrotum); T1, T2 (tail).

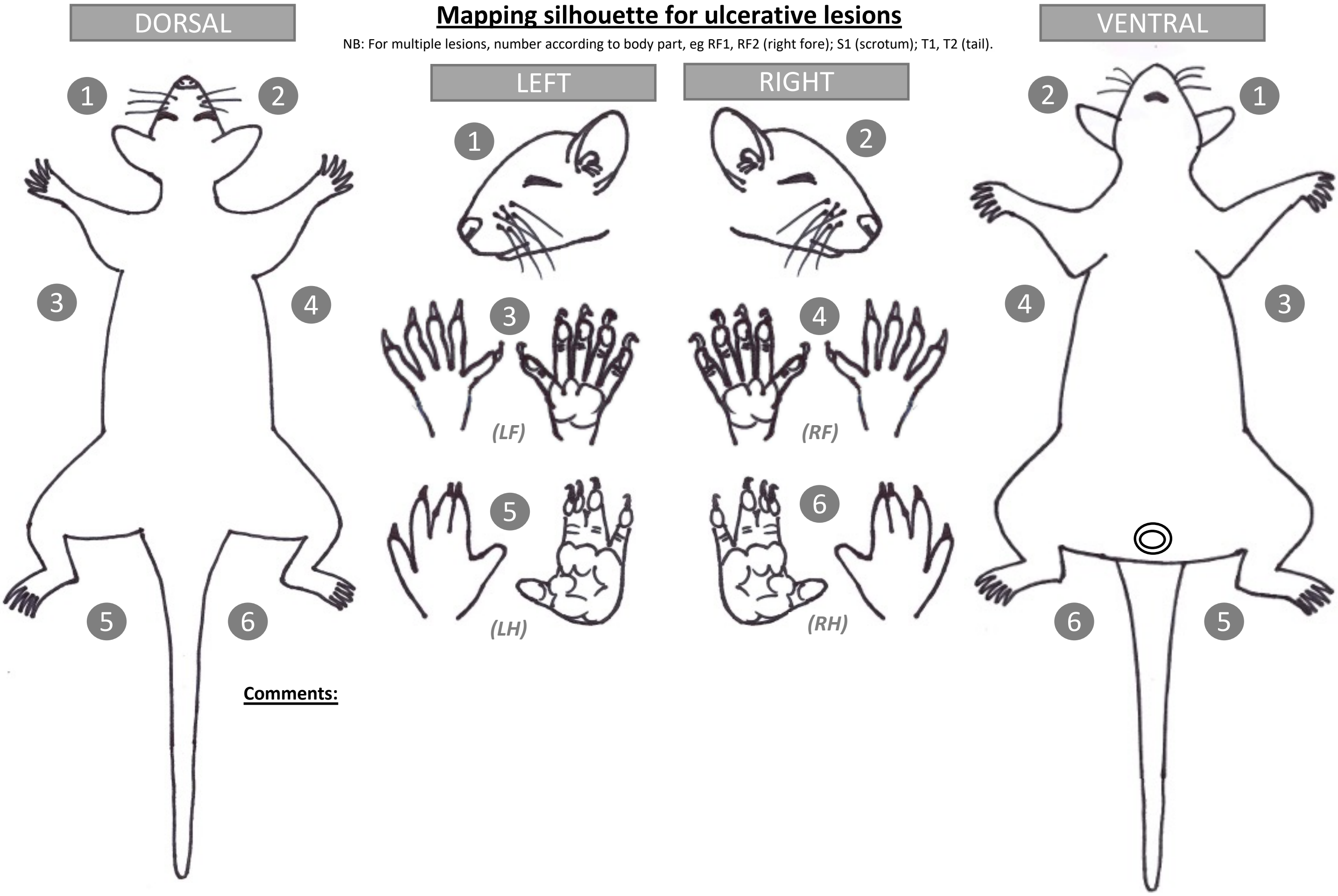

Comments:

**Welfare impacts of lesions**	[ ] **MILD** - single or multiple small lesions restricted to non-critical sites eg the tail<br>[ ] **MODERATE** - larger single lesions or multiple small lesions that involve critical site/s eg face, genitalia or footpads<br>[ ] **SEVERE** - very large single or multiple large lesions at critical sites, and any lesions exposing bone or joints

NOTES FOR REFERENCE (from previous page) (Jackson 2007; Pahl 1987)

Female reproductive status

- Non-parous: has never bred. Pouch small, clean & dry with no skin folds; very small teats.
- Parous: has bred before but not currently. Pouch small & dry but distinct & dirty; slightly elongated teats.
- Pregnant: pouch pink, possibly with skin folds visible on lateral margins.
- Pouch young present: joeys in pouch; may be attached to teat.
- Lactating: young not in pouch but still suckling. Pouch large with flaccid skin folds; hair sparse & stained; skin smooth & dark pink; teats elongated.
- Post-lactation: teats expressing only clear liquid &/or regressing.

Male reproductive status

Testis dimensions

- Sub-adult: L= 10.5mm; W= 5.8mm ; Adult: L= 23.2mm; W= 16.5mm

Sternal scent gland activity (Brushtails only): Based on absence/presence of waxy glandular product; extent of hair loss over & staining of hair surrounding gland area

- Little/no activity ; Medium level activity ; High activity

Position & area of dentine (shaded) exposed in 7 classes of tooth wear of the first molar of the upper jaw

Tooth wear class	BRUSHTAIL POSSUMS		RINGTAIL POSSUMS	
	Diagram	Age (years)	Diagram	Age (years)
1		0.9		1-1.5
2		1.2		0.5-1.2
3		1.7		0.5-3
4		3.7		1-3.5
5a		5.6		1.5-4
5b		6.8		
6a		8.9		2-5
6b		10.7		
7		11.4		2.5-4
