# Supplementary material for: Buruli ulcer surveillance in south-eastern Australian possums: Infection status, lesion mapping and internal distribution of Mycobacterium ulcerans
Source: PLoS Negl Trop Dis. 2024 Nov 5;18(11):e0012189. doi: 10.1371/journal.pntd.0012189 (PMC11581399; doi:10.1371/journal.pntd.0012189)
Supplement: S2 Appendix — S2.1 Table. IS2404 PCR results from mosquitoes opportunistically collected during Essendon possum trapping, December 2022. S2.2 Table. Statistical comparison of possums with at least one PCR positive sample (‘clinical BU’ and infected’) and with no PCR positive samples (‘uninfected’). (DOCX) [file pntd.0012189.s002.docx]

# S2 Appendix. Additional results tables.

## S2.1 Table. IS*2404* PCR results from mosquitoes opportunistically collected during Essendon possum trapping, December 2022.

| **Sample ID** | **Ct Replicate 1** | **Ct Replicate 2** | **Ct average** | **Result outcome** |
| --- | --- | --- | --- | --- |
| Mosquito 1 | 33.4 | 34.5 | 33.9 | Positive |
| Mosquito 2 | 36.9 | 37.1 | 37.0 | Positive |
| Mosquito 3 | Negative | Negative | N/A | Negative |
| Mosquito 4 | Negative | Negative | N/A | Negative |
| Mosquito 5 | Negative | Negative | N/A | Negative |
| Mosquito 6 | 39.4 | Negative | N/A | Equivocal |
| Mosquito 7 | Negative | Negative | N/A | Negative |

**S2.2 Table. Statistical comparison of possums with at least one PCR positive sample (‘clinical BU’ and ‘infected’) and with no PCR positive samples (‘uninfected’).**

| **Possum species** | **PCR positive** | **PCR negative** | **Totals** | **Odds** | **Odds ratio  (95% CI)** | **P value** |
| --- | --- | --- | --- | --- | --- | --- |
| CRT | 16 | 4 | 20 | 4 | 8 (1.06-60.33) | 0.051 |
| CBT | 2 | 4 | 6 | 0.5 |  |  |
| TOTALS | 18 | 8 | 26 |  |  |  |

CRT= common ringtail possum; CBT= common brushtail possum; CI= confidence interval
